# The diagnostic value of biomarkers (SteatoTest) for the prediction of liver steatosis

**DOI:** 10.1186/1476-5926-4-10

**Published:** 2005-12-23

**Authors:** Thierry Poynard, Vlad Ratziu, Sylvie Naveau, Dominique Thabut, Frederic Charlotte, Djamila Messous, Dominique Capron, Annie Abella, Julien Massard, Yen Ngo, Mona Munteanu, Anne Mercadier, Michael Manns, Janice Albrecht

**Affiliations:** 1 Department of Hepato-Gastroenterology, Groupe Hospitalier Pitié-Salpêtrière, Paris, France; 2 Department of Hepato-Gastroenterology, Hôpital Antoine Béclère, Clamart, France; 3 Department of Pathology, Groupe Hospitalier Pitié-Salpêtrière, Paris, France; 4 Department of Biochemistry, Groupe Hospitalier Pitié-Salpêtrière, Paris, France; 5 Department of Pathology, Hôpital Antoine Béclère, Clamart, France; 6 Department of Biochemistry, Hôpital Antoine Béclère, Clamart, France; 7 Biopredictive, Paris, France; 8 Tranfusion Unit, Groupe Hospitalier Pitié-Salpêtrière, Paris, France; 9 Division of Gastroenterology and Hepatology, Medical School of Hannover, Hannover, Germany; 10 Schering Plough Research Institute, Kenilworth NJ, USA

## Abstract

**Background:**

Biopsy is the usual gold standard for liver steatosis assessment. The aim of this study was to identify a panel of biomarkers (SteatoTest), with sufficient predictive values, for the non-invasive diagnosis of steatosis in patients with or without chronic liver disease. Biomarkers and panels were assessed in a training group of consecutive patients with chronic hepatitis C and B, alcoholic liver disease, and non-alcoholic fatty liver disease, and were validated in two independent groups including a prospective one. Steatosis was blindly assessed by using a previously validated scoring system.

**Results:**

310 patients were included in the training group; 434 in three validation groups; and 140 in a control group. SteatoTest was constructed using a combination of the 6 components of FibroTest-ActiTest plus body mass index, serum cholesterol, triglycerides, and glucose adjusted for age and gender. SteatoTest area under the ROC curves was 0.79 (SE = 0.03) in the training group; 0.80 (0.04) in validation group 1; 0.86 (0.03) in validation group 2; and 0.72 (0.05) in the validation group 3 – all significantly higher than the standard markers: γ-glutamyl-transpeptidase or alanine aminotransferase. The median SteatoTest value was 0.13 in fasting controls; 0.16 in non-fasting controls; 0.31 in patients without steatosis; 0.39 in grade 1 steatosis (0–5%); 0.58 in grade 2 (6–32%); and 0.74 in grade 3–4 (33–100%). For the diagnosis of grade 2–4 steatosis, the sensitivity of SteatoTest at the 0.30 cut-off was 0.91, 0.98, 1.00 and 0.85 and the specificity at the 0.70 cut-off was 0.89, 0.83, 0.92, 1.00, for the training and three validation groups, respectively.

**Conclusion:**

SteatoTest is a simple and non-invasive quantitative estimate of liver steatosis and may reduce the need for liver biopsy, particularly in patients with metabolic risk factor.

## Background

Fatty liver or hepatic steatosis is defined as an excessive accumulation of fat in hepatocytes [1]. On worldwide grounds, the prevalence of steatosis is very high, and is associated with several factors such as alcohol, diabetes, overweight, hyperlipidemia, insulin resistance, hepatitis C genotype 3, abetalipoproteinemia and administration of some drugs [1-4].

Fatty liver disease involves the accumulation of triglycerides in hepatocytes, apoptosis, hepatocellular ballooning, Mallory's hyaline, necrosis of hepatocytes, lobular inflammation [5,6], small hepatic vein obliteration [7] and often fibrosis with possible progression to cirrhosis, hepatocellular cancer and liver-related death [1,4,8,9].

Non-alcoholic fatty liver disease (NAFLD) is an adaptive response of the liver to insulin resistance. The natural progression of insulin resistance and endogenous noxious insults (such as free radical production, mitochondrial dysfunction, endotoxin) which are, at least in part, related to the presence of excessive fat in the liver, can trigger the development of non-alcoholic steatohepatitis (NASH). NASH itself can induce a fibrogenic response that can result in cirrhosis [5,6].

In patients with alcoholic liver disease (ALD) [10,11], chronic hepatitis C [12], and possibly in those with hepatitis B [13], the presence of steatosis is also associated with fibrosis progression, with or without associated necroinflammatory lesions (alcoholic or viral hepatitis).

Current guidelines recommend liver biopsy as part of the management of chronic liver disease [14]. This procedure provides important information regarding the degree of liver damage, in particular the severity of necroinflammatory activity, fibrosis and steatosis [14]. Unfortunately, liver biopsy has a potential sampling error, is invasive, costly and prone to complications as well [15-19]. Up to 30% of patients experience pain following the procedure; 0.3% have severe complications; and mortality approaches 0.01% [20,21].

As a result of those limitations as well as patient reluctance to undergo liver biopsy, the estimate of liver injury using non-invasive biomarkers has gained a growing importance [20-22]. For the diagnosis of fibrosis, FibroTest (FT) (Biopredictive, Paris France) has been validated as a surrogate marker in chronic hepatitis C [23] and B [24] and, recently, in ALD [25,26]. A preliminary study has also observed a similar diagnostic value in NAFLD [27]. ActiTest (AT) (Biopredictive, Paris France) has been validated as a surrogate marker for necrosis in chronic hepatitis C [23] and B [24]. Nonetheless, and despite those tests, biopsy was still useful for the diagnosis of steatosis and steatohepatitis.

For the diagnosis of steatosis, there is no standard recommendation. The usual recommendation is to measure γ-glutamyl-transpeptidase (GGT) and alanine aminotransferase (ALT) and, in addition, to perform liver biopsy for grading and staging [1,3,4,14]. The evaluation of liver steatosis using ultrasonography is subjective as based on echo intensity (echogenicity) and special patterns of echoes (texture) and is inaccurate in patients with advanced fibrosis [28]. Up to now, no study has demonstrated that a single or a panel of biomarkers can be used as an alternative to liver biopsy for the diagnosis of steatosis, whether induced by alcohol, viral hepatitis or NAFLD, the most common causes of steatosis.

The objective of the current study was to create a new panel of biomarkers known as SteatoTest (ST) with sufficient predictive values for the diagnosis of steatosis due to alcohol, NAFLD and hepatitis C and B. Serum GGT and ALT were considered as the standard biochemical markers [3].

## Results

### Patients

**Figure 1:**
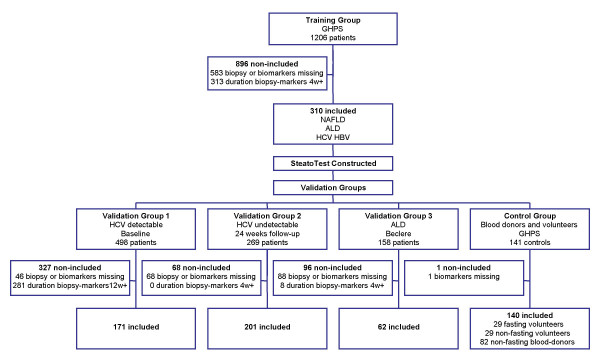
Flow chart of patients analyzed and included in the training and validation groups.

A total of 2,272 subjects were analyzed (Figure 1), being 884 subjects included in the biomarker validation study, distributed as follows: 310 patients in the training group; 171 in the validation group 1; 201 in the validation group 2; 62 in the validation group 3; and 140 subjects in the control group. The 1,388 non-included patients were not significantly different from the 884 patients integrated in the validation assay (data not shown).

### Comparison between groups (Table 1)

Patients included in the 4 groups were similar in age with a predominance of male subjects (range 61–76%). The prevalence of steatosis greater than 5% (grades 2 to 4) varied from 11% in hepatitis C virus (HCV) cured patients to 94% in patients with ALD. In all groups, at least one metabolic risk factor was observed in more than 50% of included patients. Patients in group 3 with alcoholic liver disease were more often male, older, had smaller liver biopsies, more metabolic risk factors, more extensive fibrosis and more grades 2–4 steatosis than the three other groups. Validation group 2 with HCV cured patients had quasi-normal characteristics with normal liver tests and only 11% grade 2–4 steatosis.

**Table 1:** Characteristics of the patients.

**Characteristics**	**Training group**	**Validation Group 1 – HCV before treatment**	**Validation Group 2 – HCV sustained responders**	**Validation Group 3 – Alcoholic liver disease**
Number of patients	310	171	201	62
Age at biopsy, years	48.9 (12.4)	44.1 (7.2)	43.6 (8.0)	46.6 (9.8)
Male	201 (65%)	111 (65%)	122 (61%)	47/62 (76%)
Female	109 (35%)	60 (35%)	79 (39%)	15 (24%)
BMI, kg/m^2^	25.4 (5.1)	27.7 (5.0)	26.5 (4.8)	24.2 (4.1)
**Biopsy quality**				
Length	17.0 (6.2)	16.6 (15.5)	17.0 (8.2)	13.5 (6.8)
Length ≥ 15 mm	205 (67%)	82 (48%)	96 (48%)	15 (24%)
Number of fragments	2.5 (2.3)	-	-	1.9 (1.6)
One fragment	128/278 (46%)	-	-	37 (60%)
Duration biopsy-serum, mean (days range)	1 (0–30)	40 (0–90)	11 (0–45)	7 (0–14)
**Liver Risk factor**				
HCV	211 (68%)	171 (100%)	0 (0%)	0 (0%)
HBV	18 (6%)	0 (0%)	0 (0%)	0 (0%)
NAFLD	69 (22%)	0 (0%)	0 (0%)	0 (0%)
ALD	12 (4%)	0 (0%)	0 (0%)	0 (0%)
Daily alcohol = 50 g/day	34/236 (14%)	0 (0%)	0 (0%)	62 (100%)
Cured HCV infection	0 (0%)	0 (0%)	201 (100%)	0 (0%)
**Metabolic factor**				
BMI ≥ 27.0	92 (30%)	88 (51%)	77 (38%)	14 (23%)
Glucose ≥ 6.0 mmol/L	63 (20%)	30 (18%)	27 (13%)	20 (32%)
Triglycerides ≥ 1.7 mmol/L	67 (22%)	36 (21%)	54 (27%)	20 (32%)
Cholesterol ≥ 6.0 mmol/L	61 (20%)	12 (7%)	26 (13%)	23 (37%)
**Metabolic factor: number per patient**				
None	132 (43%)	60 (35%)	96 (48%)	17 (27%)
One	101 (33%)	64 (37%)	72 (36%)	20 (32%)
Two	52 (17%)	39 (23%)	31 (15%)	19 (31%)
Three	22 (7%)	8 (5%)	0 (0%)	5 (8%)
Four	3 (1%)	0 (0%)	2 (1%)	1 (2%)
**Liver steatosis grade**				
None (0%)	130 (42%)	58 (34%)	116 (58%)	2 (3%)
Mild (Score 1–5%)	40 (13%)	68 (40%)	63 (31%)	2 (3%)
Moderate (Score 6–33%)	69 (22%)	35 (20%)	17 (8%)	42 (68%)
Marked (Score 34–66%)	36 (12%)	7 (4%)	4 (3%)	12 (19%)
Severe (Score 67–100%)	35 (11%)	3 (2%)	1 (0.5%)	4 (7%)
**Liver fibrosis stage at biopsy**				
F0 – No fibrosis	62 (20%)	0 (0%)	16 (8%)	8 (13%)
F1 – Fibrosis without septa	127 (41%)	102 (60%)	136 (68%)	23 (37%)
F2 – Few septa	52 (17%)	39 (23%)	33 (16%)	11 (18%)
F3 – Many septa	36 (11%)	19 (11%)	9 (4%)	7 (11%)
F4 – Cirrhosis	33 (11%)	11 (6%)	7 (3%)	13 (21%)
**Markers (normal range)**				
AST, IU/L (17–27 female; 20–32 male)	83 (159)	82 (57)	23 (9)	89 (83)
ALT, IU/L (11–26 female; 16–35 male)	109 (114)	118 (94)	19 (10)	72 (88)
Total bilirubin, mol/L (1–21)	14.8 (26.2)	11.1 (4.8)	8.8 (4.6)	21.5 (19.6)
GGT, U/L (7–32 female; 11–49 male)	112 (183)	84 (96)	21 (18)	323 (443)
A2M, g/L (female 1·6-4·0; male 1·4-3·3)	2.4 (1.0)	3.1 (1.2)	2.0 (0.8)	1.8 (0.5)
ApoA1 g/L (1·2-1·7)	1.4 (0.3)	1.3 (0.3)	1.2 (0.3)	1.5 (0.5)
Haptoglobin, g/L (0·35-2·00)*	0.95 (0.57)	0.78 (0.45)	0.86 (0.43)	1.39 (0.63)
Glucose, mmol/L	5.5 (3.2)	5.4 (1.2)	5.3 (1.0)	5.8 (1.6)
Cholesterol, mmol/L	4.9 (1.3)	4.5 (1.0)	5.0 (1.0)	5.4 (1.9)
Triglycerides, mmol/L	1.5 (1.4)	1.4 (0.8)	1.6 (1.0)	1.9 (3.1)
FibroTest	0.42 (0.28)	0.47 (0.26)	0.29 (0.20)	0.43 (0.28)
SteatoTest	0.49 (0.25)	0.53 (0.22)	0.36 (0.22)	0.58 (0.25)

Data are mean (SD) or proportion. BMI = body mass index; HCV = hepatitis C virus; HBV = hepatitis B virus; NAFLD = non-alcoholic fatty liver disease; ALD = alcoholic liver disease; AST = aspartate aminotransferase; ALT = alanine aminotransferase; GGT = γ-glutamyl transpeptidase; A2M = α_2_-macroglobulin; ApoA1 = apolipoprotein A1.

### Factors associated with steatosis (Table 2)

In the training group the most significant components associated with the presence of grade 2–4 steatosis in univariate analysis were body mass index (BMI), age, ALT, aspartate aminotransferase (AST), GGT, glucose, and triglycerides. The logistic regression defining the ST included 12 components – ALT, α_2_-macroglobulin (A2M), apolipoprotein A-I (ApoA1), haptoglobin, total bilirubin, GGT, cholesterol, triglycerides, glucose, age, gender and BMI. In logistic regression analyses, the most significant components were BMI (P = 0.0002), GGT (P = 0.002), ApoA1 (P = 0.01), A2M (P = 0.02), ALT (P = 0.03) and triglycerides (P = 0.04). In the validation group, similar differences were observed, most significantly for BMI, GGT, ALT and triglycerides (Table 2).

**Table 2:** Characteristics of the patients, according to the presence of steatosis.

**Characteristic**	**Steatosis Training Group**			**Steatosis Validation Group 1 – HCV before treatment**		
	**< 5%, n = 170**	**≥ 5%, n = 140**	**P value**	**No, n = 126**	**Yes, n = 45**	**P value**

**Demographics**						
Age at biopsy, years	46.7 (12.4)	51.8 (12.1)	0.0004	43.7 (7.3)	45.2 (7.0)	0.28
Male gender	110 (55%)	91 (45%)	0.96	81 (64%)	30 (67%)	0.77
BMI	24 (4)	27 (6)	< 0.0001	27 (5)	31 (4)	< 0.0001
**Biochemical markers**						
α_2_-macroglobulin, g/L	2.47 (1.00)	2.30 (1.04)	0.07	3.10 (1.23)	3.20 (1.24)	0.50
ALT, IU/L	104 (119)	115 (108)	0.02	46 (45)	61 (48)	0.003
AST, IU/L	83 (204)	83 (78)	0.01	80 (61)	88 (43)	0.01
Apolipoprotein A1, g/L	1.46 (0.34)	1.42 (0.33)	0.30	1.27 (0.26)	1.20 (0.24)	0.18
Haptoglobin, g/L	0.93 (0.60)	0.96 (0.52)	0.19	0.77 (0.45)	0.78 (0.44)	0.84
GGT, IU/L	83 (132)	147 (226)	< 0.0001	72 (85)	118 (116)	0.0007
Total bilirubin, μmol/L	14.8 (31.4)	14.7 (17.8)	0.47	11.0 (5.0)	11.3 (4.1)	0.38
Glucose mmol/L	5.1 (3.7)	5.9 (2.2)	< 0.0001	5.2 (0.9)	6.0 (1.8)	0.0007
Triglycerides, mmol/L	1.24 (0.95)	1.88 (1.78)	< 0.0001	1.26 (0.72)	1.72 (1.0)	0.0008
Total cholesterol, mmol/L	4.8 (1.2)	5.1 (1.4)	0.10	4.5 (1.0)	4.4 (1.0)	0.10
FibroTest	0.40 (0.29)	0.45 (0.28)	0.47	0.45 (0.26)	0.53 (0.24)	0.07
SteatoTest	0.38 (0.21)	0.62 (0.22)	< 0.0001	0.47 (0.21)	0.70 (0.16)	< 0.0001

**Characteristic**	**Steatosis Validation Group 2 – HCV sustained responders**			**Steatosis Validation Group 3 – Alcoholic liver disease**		

	**No n = 179**	**Yes n = 22**	**P value**	**< 5%, n = 4**	**≥ 5%, n = 58**	**P value**

**Demographics**						
Age at biopsy, years	43.7 (8.1)	43.1 (7.0)	0.7	38.0 (12.8)	47 (9.4)	0.16
Male gender	110 (62%)	12 (55%)	0.53	2 (50%)	45 (78%)	0.21
BMI	26 (4)	31 (6)	<0.0001	22.9 (2.9)	24.3 (4.2)	0.49
**Biochemical markers**						
α_2_-macroglobulin, g/L	2.08 (0.79)	1.73 (0.66)	0.06	2.12 (0.53)	1.81 (0.55)	0.26
ALT, IU/L	18 (9)	26 (9)	<0.0001	35 (24)	74 (90)	0.10
AST, IU/L	23 (9)	25 (7)	0.06	74 (43)	58 (90)	1.00
Apolipoprotein A1, g/L	1.16 (0.28)	1.07 (0.25)	0.2	1.67 (0.43)	1.48 (0.49)	0.49
Haptoglobin, g/L	0.85 (0.41)	0.94 (0.56)	0.85	1.55 (0.92)	1.38 (0.62)	0.85
GGT, IU/L	20 (18)	28 (14)	0.0002	327 (184)	323 (323)	0.41
Total bilirubin, μmol/L	8.9 (4.6)	8.1 (4.3)	0.3	28.5 (23.4)	21.1 (19.5)	0.28
Glucose, mmol/L	5.3 (1.0)	5.5 (0.8)	0.16	6.5 (2.2)	5.7 (1.6)	0.46
Triglycerides, mmol/L	1.49 (0.98)	2.05 (1.22)	0.003	1.05 (0.51)	1.96 (3.15)	0.28
Total cholesterol, mmol/L	5.0 (1.0)	5.1 (0.9)	0.51	6.0 (1.38)	5.4 (2.0)	0.68
FibroTest	0.29 (0.20)	0.26 (0.19)	0.46	0.43 (0.32)	0.43 (0.28)	0.79
SteatoTest	0.32 (0.20)	0.62 (0.17)	<0.0001	0.44 (0.03)	0.59 (0.26)	0.21

Data are mean (SD) or proportion.

### Distribution of SteatoTest according to steatosis grades (Figure 2)

**Figure 2:**
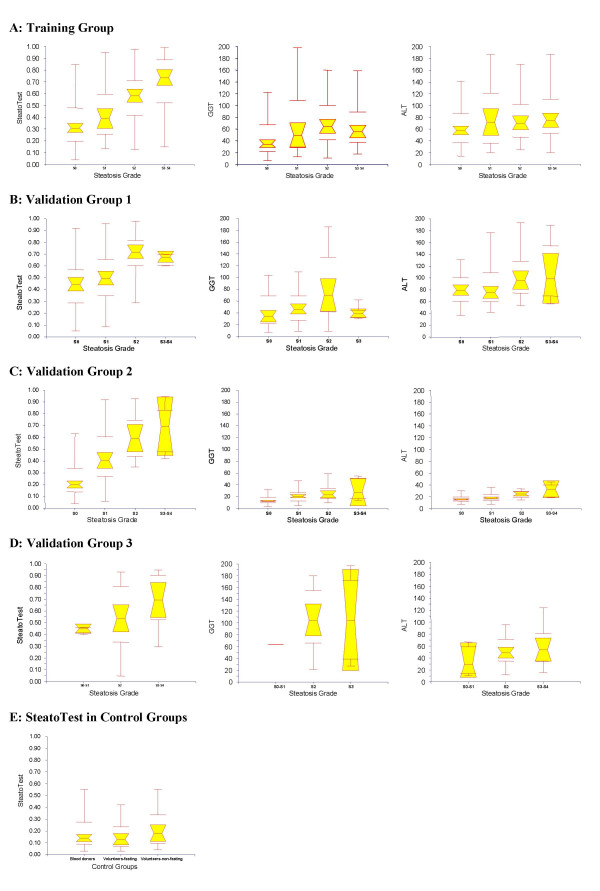
**Relationship between ST, GGT and ALT and the grade of liver steatosis. **A four grades scoring system was used to assess steatosis: S0 – no steatosis; S1 – mild, 1 to 5%; S2 – moderate, 6 to 32%; S3-S4 – marked or severe, 33 to 100%. Notched box plots showing the relationship (A) in the training group; (B) in validation group 1, HCV patients before treatment; (C) group 2, HCV sustained responders; (D) group 3, alcoholic liver disease; and (E) in controls, healthy volunteers fasting and non-fasting and non-fasting blood donors. The horizontal line inside each box represents the median and the width of each box the median ± 1.57 interquartile range/vn for assessing the 95% level of significance between group medians. Failure of the shaded boxes to overlap corresponds to statistical significance (P < 0.05). The horizontal lines above and below each box encompass the interquartile range (from 25^th ^to 75^th ^percentile), and the vertical lines from the ends of the box encompass the adjacent values (upper: 75^th ^percentile plus 1.5 times interquartile range, lower 25^th ^percentile minus 1.5 times interquartile range). In validation group 3, almost all patients had steatosis and group S0 and S1 were combined.

The median ST value was 0.13 in fasting controls; 0.18 in non-fasting controls; 0.14 in blood donors; 0.26 in patients without steatosis; 0.43 in grade 1 steatosis; 0.62 in grade 2; 0.70 in grade 3; and 0.75 in grade 4. Because there were not a sufficient number of patients with grade 3 and 4, these two groups were combined (Figure 2).

### Diagnostic value of SteatoTest (Tables 3 and 4)

The values {Area under the ROC curves (AUROCs)} of ST, GGT and ALT for the diagnosis of grades 2–4 steatosis, in the training and validation groups, are given in Table 3. ST had higher AUROCs: {0.79 (SE = 0.03)} in training group; 0.80 (0.04) in validation group 1; 0.86 (0.03) in validation group 2; and 0.72 (0.05) in validation group 3. These were always significantly higher than the AUROCs of GGT and significantly higher than the AUROCs of ALT, for the training group and validation group 1 (Table 3). The distribution of ST, GGT and ALT, according to the severity of steatosis, is illustrated in Figure 2 for the training and validation groups.

**Table 3:** Values {Area under the ROC curves (AUROCs)} of SteatoTest, GGT and ALT for the diagnosis of steatosis greater than 5%, in both training and validation groups.

**Diagnostic panel**	**Training Group AUROC (se)**	**Validation Group 1 – HCV before treatment**	**Validation Group 2 – HCV sustained responders**	**Validation Group 3 – Alcoholic liver disease**	**All groups**
	N = 310	N = 171	N = 201	N = 62	N = 884
SteatoTest	0.79 (0.03)*	0.80 (0.04)£	0.86 (0.03) $	0.72 (0.05)**	0.80 (0.02) ££
GGT	0.66 (0.03)	0.67 (0.05)	0.74 (0.05)	0.50 (0.09)	0.66 (0.02)
ALT	0.58 (0.03)	0.62 (0.05)	0.79 (0.04)	0.66 (0.07)	0.61 (0.02)

* – Higher than GGT (P < 0.0001) and ALT (P < 0.0001); £ – Higher than GGT (P = 0.007) and ALT (P < 0.0001); $ – Higher than GGT (P = 0.02); ** – Higher than GGT (P = 0.002); ££ Higher than GGT (P < 0.0001) and ALT (P < 0.0001).

The diagnostic values of ST, GGT and ALT according to cutoffs are shown in Table 4. For the diagnosis of grade 2–4 steatosis, the sensitivity of ST at the 0.30 cut-off was 0.91, 0.98, 1.00 and 0.85 and the specificity at the 0.70 cut-off was 0.89, 0.83, 0.92, and 1.00, for the training and validation groups, respectively.

**Table 4:** Diagnostic value of SteatoTest for predicting liver steatosis greater than 5%.

**Cut-off**	**Sensitivity**	**Specificity**	**Positive Predictive Value**	**Negative Predictive Value**
**Training Group N = 310**			Prevalence = 45%	
SteatoTest 0.30	0.91 (127/140)	0.45 (77/170)	0.58 (127/220)	0.86 (77/90)
SteatoTest 0.50	0.69 (97/140)	0.74 (126/170)	0.69 (97/141)	0.75 (126/169)
SteatoTest 0.70	0.45 (63/140)	0.89 (152/170)	0.78 (63/81)	0.66 (152/229)
GGT 50 IU/L	0.66 (92/140)	0.55 (94/170)	0.55 (92/168)	0.66 (94/142)
ALT 50 IU/L	0.77 (108/140)	0.35 (60/170)	0.50 (108/218)	0.65 (60/92)
**Validation Group1 N = 171**			Prevalence = 26%	
SteatoTest 0.30	0.98 (44/45)	0.24 (30/126)	0.31 (44/140)	0.97 (30/31)
SteatoTest 0.50	0.89 (40/45)	0.58 (73/126)	0.43 (40/93)	0.94 (73/78)
SteatoTest 0.70	0.44 (20/45)	0.83 (105/126)	0.49 (20/41)	0.81 (105/130)
GGT 50 IU/L	0.62 (28/45)	0.61 (72/126)	0.34 (28/82)	0.81 (72/89)
ALT 50 IU/L	1.00 (45/45)	0.06 (8/126)	0.28 (45/163)	1.00 (8/8)
**Validation Group 2 N = 201**			Prevalence = 11%	
SteatoTest 0.30	1.00 (22/22)	0.56 (100/179)	0.22 (22/101)	1.00 (100/100)
SteatoTest 0.50	0.68 (15/22)	0.79 (142/179)	0.29 (15/52)	0.95 (142/149)
SteatoTest 0.70	0.32 (7/22)	0.92 (165/179)	0.33 (7/21)	0.92 (165/180)
GGT 50 IU/L	0.09 (2/22)	0.97 (174/179)	0.29 (2/7)	0.90 (174/194)
ALT 50 IU/L	0.05 (1/22)	0.98 (176/179)	0.25 (1/3)	0.89 (176/197)
**Validation Group 3 N = 62**			Prevalence = 94%	
SteatoTest 0.30	0.85 (49/58)	0.00 (0/4)	0.93 (49/53)	0.00 (0/9)
SteatoTest 0.50	0.62 (36/58)	1.00 (4/4)	1.00 (36/36)	0.15 (4/26)
SteatoTest 0.70	0.40 (23/58)	1.00 (4/4)	1.00 (23/23)	0.10 (4/39)
GGT 50 IU/L	0.90 (52/58)	0.00 (0/4)	0.93 (52/56)	0.00 (0/6)
ALT 50 IU/L	0.53 (31/58)	0.75 (3/4)	0.97 (31/32)	0.10 (3/30)
**All Groups N = 884**			Prevalence = 30%	
SteatoTest 0.30	0.90 (238/265)	0.54 (336/619)	0.46 (238/521)	0.93 (336/363)
SteatoTest 0.50	0.72 (190/265)	0.75 (466/619)	0.55 (190/343)	0.86 (466/541)
SteatoTest 0.70	0.46 (122/265)	0.88 (546/619)	0.63 (122/195)	0.79 (546/689)
GGT 50 IU/L	0.66 (174/265)	0.76 (468/619)	0.54 (174/325)	0.84 (468/559)
ALT 50 IU/L	0.72 (185/265)	0.62 (382/619)	0.44 (185/422)	0.83 (382/462)

In the training group, there were 56 cases (18%) of significant discordance between steatosis percentage as predicted by ST and that observed in biopsy samples. Failure attributable to ST (false positive of ST) was suspected in one case that had acute drug hepatitis associated with chronic hepatitis B. Failure attributable to biopsy (false negatives of biopsy) was suspected in 16 cases with poor quality biopsy samples (median length 13 mm, 2 fragments) and, at least, one metabolic risk factor. For the validation' groups, significant discordance was observed in 17 cases (16%) in group 1; 20 cases (10%) in group 2; and 13 cases (21%) in group 3. Significant discordance was observed more often in patients with extensive fibrosis (stage F3 or F4): 38 cases out of 135 (28%) *versus *91 cases out of 609 (15%) – P = 0.001.

### Repeated biopsies and repeated SteatoTest

A total of 75 patients were included with biopsy at baseline and at follow-up. Among them, 23 had an improvement of steatosis (one of 3 grades, two of 2 grades and twenty of one grade); 43 had no change in steatosis grade; and 9 had worsening of one grade. ST significantly decreased in 23 patients with steatosis improvement at biopsy from 0.60 (SE = 0.05) to 0.41 (0.05), a significantly greater difference (P = 0.001) than that observed in 52 patients without biopsy improvement: from 0.44 (0.03) to 0.31 (0.03).

### Integrated database

**Figure 3:**
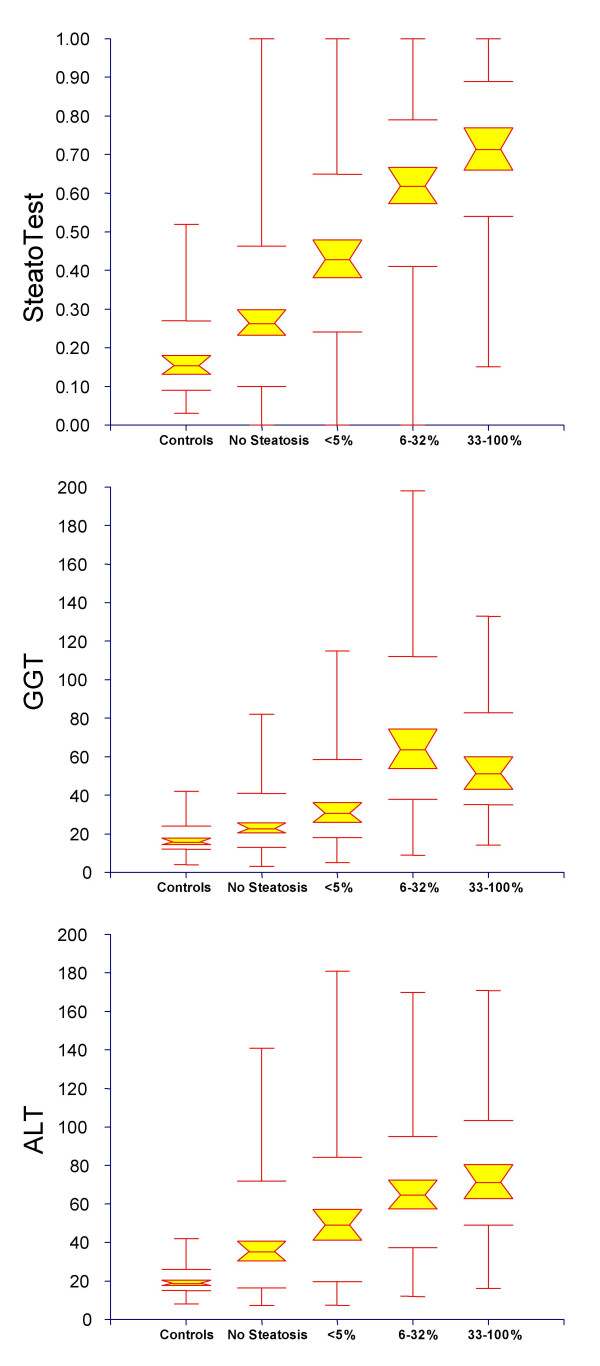
**Relationship between ST, and the grade of liver steatosis in the integrated database combining controls, training group and validation groups. **Failure of the shaded boxes to overlap indicates statistical significance between medians (P < 0.05). There was a significant difference between all grades by the Tukey-Kramer multiple comparison test for all pairwise differences between means (P < 0.05). For GGT and ALT, there was no significant difference between S0 and S1 and between S2 and S3. For ALT, there was also no significant difference between S0 and S2, S1 and S2.

A total of 884 subjects were included in the integrated database combining the training group, the three validation groups and the control group. Of these, 75 patients with HCV were investigated twice (once before and then after treatment), and 29 volunteers were investigated twice (while fasting and, then, non-fasting). There was a very significant overall correlation between ST and the steatosis grades from controls to S3 (Figure 3). For ST, there was a significant difference between all histological grades by Tukey-Kramer multiple comparison test for all pairwise differences between means (P < 0.05). For GGT and ALT, there was no significant difference between S0 and S1. For ALT, there was no significant difference between S0 and S2, S1 and S2, and S2 and S3, either. ST has higher AUROC, 0.80 (0.02) than all the isolated components for the diagnosis of steatosis grade 2–4: ALT, GGT (Table 3), triglycerides 0.63 (0.02), BMI 0.61 (0.02), glucose 0.61 (0.02), bilirubin 0.60 (0.02), ApoA1 0.56 (0.02), A2M 0.56 (0.02) and cholesterol 0.53 (0.02) – all P values < 0.03.

A cut-off of 0.30 had 90% sensibility and a cut-off of 0.70 had 88% specificity permitting to achieve useful predictive values for steatosis grade 2–4, 93% negative predictive value (NPV) and 63% positive predictive value (PPV) for a steatosis prevalence of 30% (Table 4). The 90% specificity was obtained for a 0.72 cut-off with a corresponding 63% PPV. The overall percentage of patients classified with at least 90% sensitivity or 90% specificity was 59% (363+156/884).

Among the 744 patients with biopsy, for the diagnosis of steatosis 3–4, the ST AUROC was 0.79 (0.02), significantly higher than GGT 0.74 (0.02) (P = 0.03), and ALT was 0.71 (0.02) (P = 0.007). The 90% sensitivity was obtained for a 0.32 cut-off; the 90% specificity was obtained for a 0.81 cut-off.

### Conversion between SteatoTest results and the corresponding steatosis grade

ST is a continuous linear biochemical assessment of steatosis grade. It provides a numerical quantitative estimate of liver steatosis ranging from 0.00 to 1.00, corresponding to a steatosis scoring system of grades S0 to S4. Among the 140 controls, the median ST value (± SE) was 0.08 ± 0.004 (95th percentile, 0.23). Among the 744 patients with liver biopsy, the ST conversion was 0.000 – 0.3000 for S0; 0.3001 – 0.3800 for S0-S1; 0.3801 – 0.4800 for S1; 0.4801 – 0.5700 for S1-S2; 0.5701 – 0.6700 for S2; 0.6701 – 0.6900 for S2-S3S4; and 0.6901 – 1.000 for S3-S4.

### Steatosis at Ultrasonography and SteatoTest

Ultrasonography has been preformed together with ST and biopsy in 304 patients. Concordance between steatosis diagnosed, at ultrasonography and at biopsy, was lower (kappa coefficient = 0.32 ± 0.05) than the concordance with ST (at 0.50 cut-off, kappa = 0.44 ± 0.06; P = 0.02), as well as lower AUROC 0.65 ± 0.03 for ultrasonography *versus *0.78 ± 0.03 for ST (P = 0.001). The ST values according to the presence of histological and radiological steatosis are given in Table 5.

**Table 5:** SteatoTest value according to presence of liver steatosis greater than 5% at liver biopsy, and according to presence at ultrasonography.

	**No steatosis at biopsy**	**Steatosis at biopsy**	**Significance**
**No steatosis at ultrasonography**	N = 143, ST = 0.37± 0.02	N = 74, ST = 0.55± 0.02	< 0.0001
**Steatosis at ultrasonography**	N = 25, ST = 0.47± 0.04	N = 62, ST = 0.70± 0.03	< 0.0001
**Significance**	0.01	< 0.0001	

### Sensitivity analyses

A total of 635 (85%) patients had a time lapse between biopsy and serum smaller than one month. The AUROC of ST was similar in those patients (0.77, 95% CI 0.73–0.80) than in the 109 (15%) patients with greater lapse (0.82, 95% CI 0.72–0.89; P = 0.36). A total of 670 (78%) patients had a biopsy sample length smaller than 20 mm. The AUROC of ST was slightly smaller in those patients (0.76, 95% CI 0.71–0.79) than in the 161 (15%) patients with greater sample (0.82, 95% CI 0.74–0.88; P = 0.10).

## Discussion

Our results highlight the utility of a new panel of biochemical markers (ST) for the prediction of steatosis of different origins. A cut-off of 0.30 had 90% sensibility and a cut-off of 0.72 had 90% specificity permitting to achieve useful predictive value, 93% NPV and 63% PPV for a steatosis prevalence of 30%. These predictive values are far from perfection, particularly for PPV; however, already predictive and significantly higher than those of previous usual markers GGT, ALT and ultrasonography, as demonstrated by the increase of AUROCs. This benefit was observed for the most frequent chronic liver diseases: chronic viral hepatitis, and alcoholic and non-alcoholic fatty liver diseases.

We have not identified any reports of a single or a combination of biomarkers with accurate value for the diagnosis of steatosis in different chronic liver diseases. Marceau et al observed in 551 severely obese patients with liver biopsy that steatosis was associated with male gender, age, BMI, waist/hip ratio, diabetes, systolic blood pressure, fasting blood sugar, triglycerides, and non-HDL cholesterol, but no diagnostic algorithm was provided [29]. Papadia *et al*. [30] observed in 1000 obese patients an association between steatosis and AST, ALT, AST/ALT ratio, body weight, waist/hip ratio, serum glucose, serum triglycerides, BMI, GGT, age, and unconjugated bilirubin using regression analysis [30]. No panel was constructed and they concluded that no reliable biochemical marker could identify patients with severe steatosis with sufficient sensitivity for avoiding liver biopsy. Loguercio *et al*. [31] observed that in 305 patients with abnormal GGT or ALT, age, ferritin and tissue 4-hydroxynonenal were associated with steatosis. On multivariate analysis, no single factor was found to be an independent predictor [31].

In the present study, the predictive value of ST was related to the discriminant values of its different components. The most striking observation was that the combination of 12 parameters allowed a very significant increase in the diagnostic values of isolated GGT or ALT. The diagnostic value of ALT was better than that of GGT, as assessed by AUROCs in all the different groups. This is surprising as an elevated GGT is generally thought to be a serum marker of steatosis and elevated transaminases to be a marker of NASH. A better association between ALT and steatosis *versus *GGT and steatosis has also been observed using proton magnetic resonance imaging [32].

The diagnostic values of GGT, ALT, triglycerides, cholesterol, glucose and BMI were expected, because they had been previously associated with steatosis of different origins [3,29,31]. Those biomarkers are also associated with insulin resistance and triglyceride deposition in the liver [6]. ApoA1 is highly associated with HDL-cholesterol and a negative association was also expected with steatosis [29]. The advantage of combining biomarkers of steatosis and those more specific for fibrosis such as A2M, haptoglobin and bilirubin is to adjust the predictive values according to the associated stage of fibrosis. In the present study we observed that the grade of steatosis in patients with extensive fibrosis was significantly lower than in patients without extensive fibrosis (data not shown).

Our study has several limitations that must be acknowledged. Firstly, despite the use of prospective cohorts of patients, our study was not a classical prospective study. The validation groups consisted of previously studied groups of patients: groups 1 and 2 were from a prospective randomized trial with a previous publication on steatosis [33], and group 3 was a prospective cohort of patients with alcoholic liver disease from a study which had been published for validation of fibrosis biomarkers [26]. There were three different pathologists but very skilled in these scoring systems and expert in variability studies. The analyses of histological specimens and biochemical markers were performed blindly, and the recommended pre-analytical and analytical procedures were respected for most of the components. The analytical variability of cholesterol, triglycerides and glucose should be assessed.

A second limitation was the relatively small number of patients with grade 3 and 4 steatosis. We observed a non-significant difference between ST medians, 0.70 for grade 3 *versus *0.75 for grade 4. Due to the small sample size of patients with grade 3–4 steatosis in the validation groups, further studies should be performed in order to determine whether ST could discriminate between patients with marked steatosis (between 30 and 66%) and those with severe steatosis (over 66%). Grade 3 and 4 steatosis is more frequent in patients with NAFLD and further studies must be performed in these patients.

In patients with NAFLD, a liver biopsy is more usually obtained for identifying additional features of steatohepatitis (hepatocellular ballooning, lobular inflammation, Mallory's hyaline) which may be associated with and/or predictive for the development of pericellular and/or periportal fibrosis. FT has been already validated for the diagnosis of fibrosis in NAFLD [27] and ALD [26]. Studies on biomarkers of steatohepatitis (NashTest, AshTest) are also in progress (personal communication of Thierry Poynard). Combination of those non-invasive markers should help the physician in the management of NAFLD and ALD.

A third limitation was not having compared prospectively the serum biomarkers with imaging techniques such as ultrasonography [28,32,34] and proton magnetic resonance imaging [35]. In the retrospective analysis of the training population, we observed that ST had a higher diagnostic value than the routine ultrasonography with higher AUROCs. It has been already observed that the sensitivity of ultrasonography is low in obese patients [36] for the diagnosis of steatosis. Proton magnetic resonance imaging is expensive; nevertheless, a validation of ST versus proton magnetic resonance imaging would be quite interesting.

In contrast with the above mentioned limitations, one advantage of the present design was the inclusion of heterogeneous patients in the training group with different causes of chronic liver disease as well as the validation of the diagnostic values in more homogeneous groups. Validation groups 1 and 3 included very homogeneous patients, with chronic hepatitis C and ALD, respectively. The advantage of validation group 2 was the inclusion of a group of patients clinically and biologically close to a "normal" population, as these patients are sustained virologic responders and had quasi-normal liver function tests. This population offered the unique opportunity of having liver biopsies in subjects with normal profiles – not possible, for example, in blood donors. The intra and inter-laboratory variability has been studied for the 6 FT components and those studies should also be performed for cholesterol, triglycerides and glucose. We did not find any significant differences in ST AUROCs according to ethnicity (data not showed) [37].

As discussed for liver fibrosis, it is also possible that the limitations of liver biopsy (sampling error and pathologist concordance) did not allow a perfect area under the curve to be reached [38]. In hepatitis C the ideal gold standard would be at least a 40 mm length biopsy sample. Bedossa *et al*. [18] recommend, at least, 25 mm; but the coefficient of variation decreases up to 40 mm. In chronic hepatitis C, 18 % of discordance in fibrosis staging has been attributed to liver biopsy failures (mainly due to small sample size) and 2% to FT (due to hemolysis, inflammation and Gilbert's syndrome) [38]. For liver steatosis, there is also a sampling variability with discordance in 22% of patients [19]. In the present study, we observed discordance between steatosis assessed by ST and that assessed by biopsy, in 10% to 21% according to patient's group. Several discordant cases seem to be attributable to biopsy (false negatives of biopsy) as the quality was poor and, at least, one metabolic risk factor was present. Significant discordance was more often observed in patients with extensive fibrosis. We previously suspected a risk of greater variability in assessing fibrosis when steatosis was present but the inverse could be also true: a greater variability in assessing steatosis in case of cirrhotic or pre-cirrhotic stages [38].

ST is not a perfect diagnostic tool, but has several advantages over other proposed strategies for steatosis management. The 12 components of ST are readily available. FibroTest-ActiTest is now available in several different countries, including the USA (FibroSure™), with a quality charter for laboratories for reducing inter-laboratory variability [23,30,38,39]. As demonstrated in the present study, ST allowed the assessment of steatosis in patients with paired biopsy. This could be very useful for the follow-up of patients. This has been validated in HCV patients before and after treatment and should be validated in patients with ALD and NAFLD with paired biopsies.

There is no specific approved treatment for steatosis. Recommendations depend on the cause. There is wide agreement for the cessation of alcohol consumption in heavy drinkers, weight reduction in obese patients, and the treatment of diabetes and hyperlipidemia [1-4]. In patients with chronic hepatitis C and genotype 3, 50% of the patients treated and who have a sustained virologic response have a disappearance of liver steatosis at the second biopsy [33]. Bellentani *et al*. [3] recommended that subjects with elevated ALT or GGT should be screened for steatosis using hepatic ultrasonography. They suggested that the demonstration of hepatic steatosis should prompt a reduction of caloric and alcohol intake and follow-up with both ultrasonography and biochemical tests. When clinically indicated, a liver biopsy for assessing the degree of fibrosis and inflammation could be performed.

## Conclusion

According to the low predictive values of ALT, GGT and ultrasonography, as well as the risk and the variability of liver biopsy, the previous strategy could be improved by using better biomarkers of steatosis, such as ST, combined with biomarkers of fibrosis, such as FibroTest-Fibrosure, and with biomarkers of steatohepatitis. The cost will be probably similar to the price of FibroTest-Fibrosure (currently around 100 €) and cheaper than biopsy or proton magnetic resonance imaging. This new strategy will likely reduce the indications of liver biopsy. Prospective studies are needed to confirm those results and to support the general use of this new biomarker.

## Methods

### Study population

Consecutive patients who were included were those with an available serum sample, a liver biopsy, and a time interval between serum sampling and biopsy of less than three months (Figure 1).

### Training group (mixed liver diseases)

These patients were retrospectively included for this specific analysis, but had been analyzed in previous prospective validation studies of FT between September 2000 and August 2004 [23,24,27,38]. All were patients hospitalized in the of Hepato-Gastroenterology department of Groupe Hospitalier Pitié-Salpêtrière for NAFLD, hepatitis C and B, and ALD.

### Validation group one (hepatitis C)

These patients were retrospectively analyzed from a study of steatosis in patients with chronic hepatitis C [33]. For this purpose, previously non-treated patients of a prospective multicentre randomized trial of pegylated-Interferon and ribavirin were included. The biomarkers and the biopsy results at baseline were used.

### Validation group two (former hepatitis C, with undetectable HCV)

These patients were those from the patients of the same randomized trial [33] who had been "cured" – they had a sustained virologic response, with undetectable HCV RNA, at the end of treatment and 24 weeks after the end of treatment. The biomarkers and the biopsy results performed 24 weeks after the end of treatment were used. This group can be considered to be a validation group of non-viral steatosis because possible viral steatosis had been cured by the treatment [33].

### Validation group three (ALD)

These patients were retrospectively included for this specific analysis but had been prospectively included between 1998 and 2000 in a cohort of alcoholic patients for which one primary endpoint was the identification of biochemical markers. The details of this cohort have been recently published in a validation study of FT [26]. All were patients hospitalized in the Hepato-Gastroenterology Department of Hôpital Antoine Béclère, for complications of alcoholic liver disease.

### Common criteria of non-inclusion

Non-inclusion criteria included non-available serum, non-available biopsies and biopsy and serum samples which had been collected more than 3 months apart (Figure 1). Patient characteristics are given in Table 1.

### Control groups

This included a group of, apparently, healthy volunteers who had been previously included in a validation study of FT, in fasting and non-fasting conditions [39]. A group of non-fasting blood donors were also prospectively included.

### Histologic analysis

Common rules were applied to the different groups. Liver biopsy specimens were processed using standard techniques. Patients with viral hepatitis were evaluated for fibrosis and grade of activity according to the METAVIR scoring system, for which reproducibility had previously been established [40]. Patients with ALD and NAFLD were evaluated with modified staging and grading scores [41-44]. Fibrosis was staged on a scale of 0 to 4: F0 – no fibrosis; F1 – portal fibrosis or perivenular fibrosis without septa; F2 – few septa; F3 – numerous septa without cirrhosis; and F4 – cirrhosis. Activity (the intensity of necroinflammatory activity mostly based on necrosis) was scored as follows: A0 – no histologic activity; A1 – mild activity; A2 – moderate activity; and A3 – severe activity. Steatosis was scored from 0 to 4 with a four grades scoring system from S0 to S4: S0 – no steatosis; S1 – mild 1 to 5% (% of hepatocytes containing visible macrovesicular steatosis); S2 – moderate 6 to 32%; S3 – marked 33 to 66%; and S4 – severe 67 to 100% [33]. The main histological criterion was the presence of steatosis grade 2–4 (between 6 to 100%). A single pathologist per group, unaware of patient characteristics, analyzed the histological features (Frederic Charlotte for the training group, Zack Goodman for validation groups 1 and 2, and Dominique Capron for validation group 3).

### Serum biochemical markers – New biomarker of steatosis

A new panel (ST, Biopredictive, Paris, France, patent pending) was constructed in the training group combining the 6 components of the FibroTest-ActiTest (patented artificial intelligence algorithm USPTO 6,631,330) adjusted for age, gender and BMI, plus serum glucose, triglycerides and cholesterol. ST scores of range from zero to 1.00, with higher scores indicating a greater probability of significant lesions. FT and AT (Biopredictive, Paris, France; FibroSURE LabCorp, Burlington, NC, USA) were determined as has been previously published [23,38,39]. The published recommended pre-analytical and analytical procedures were used [23,38,39,45,46]. In the training and control groups, GGT, ALT, serum glucose, triglycerides, cholesterol, and total bilirubin were measured by Hitachi 917 analyzer or Modular DP analyzers (both Roche Diagnostics Mannheim, Germany) using the manufacturer's reagents. A2M, ApoA1, and haptoglobin were measured using an automatic nephelemeter BNII (Dade Behring; Marburg, Germany). In validation groups 1 and 2, GGT, ALT, serum glucose, triglycerides, cholesterol, and total bilirubin were measured using Hitachi 747 or 911 (Roche Diagnostics, Indianapolis, IN, USA) with the manufacturer's reagents. ApoA1, A2M and haptoglobin were determined in serum samples using an automatic nephelometer BNII (Dade Behring, Marburg, Germany). In validation group 3, ALT, GGT, serum glucose, triglycerides, cholesterol, total bilirubin and haptoglobin were measured by autoanalyzer (Olympus AU 640 Automate) using manufacturer's reagents (Olympus, Rungis, France); A2M and ApoA1 were measured using an automatic nephelometer (BNII, Dade Behring, Marburg, Germany). All coefficients of variation assays were lower than 10%.

### Imaging

Ultrasonography reports have been retrospectively analyzed for the presence or absence of radiological steatosis in the validation group, blindly to histological and biochemical data.

### Statistical analyses

The primary outcome was grade 2, 3 or 4 of steatosis (S2S3S4). The cause of discordance between the presence of S2S3S4 steatosis, as predicted by biochemical markers and biopsy was attributed according to respective risk factors of failure, as previously detailed [38]. Significant discordance was defined as discordance in predicting grades S2S3S4 and a 30% or greater difference in steatosis percentage, as predicted by ST or as observed in the biopsy sample. Risk factors of ST failure were hemolysis, Gilbert's disease, acute inflammation and extra-hepatic cholestasis. Risk factors of biopsy failure were biopsy size (less than 25 mm) and fragmentation (more than one fragment). Failure attributable to biopsy (false negative) was suspected when the biopsy length was less than 15 mm and fragmented with the additional presence of, at least, one metabolic risk factor.

Statistical analysis used Fisher's exact test, the chi-square test, Student's t-test and the Mann-Whitney test; variance analysis used the Bonferroni all-pair wise and the Tukey-Kramer multiple-comparison tests to take into account the multiple comparisons, and multiple logistic regression the for multivariate analysis [47]. The diagnostic values of the markers were assessed using sensitivities, specificities, PPVs and NPVs and AUROCs [47]. Corresponding steatosis grades were calculated from median ST scores and 95% confidence intervals observed in 744 patients and 140 controls. AUROCs were calculated using the empirical non-parametric method according to Delong *et al*. [48] and compared using the method of Zhou *et al*. [49]. The binomial approach was used only when the sample size population was less than 30 [50]. For all analyses, two-sided statistical tests were used; a P-value of 0.05 or less was considered significant. Number Cruncher Statistical Systems 2003 software (NCSS, Kaysville, Utah, USA) was used for all analyses [47].

A sensitivity analysis was also performed for determining the accuracy of the markers for the primary outcomes according to biopsy sample size (less than 20 mm or more) and to time lapse between serum and biopsy (less than 4 weeks or more).

## Competing interests

Thierry is the inventor of both the FT and the ST, is a consultant and has a capital interest in Biopredictive, the company marketing FibroTest-SteatoTest. Mona Munteanu is employee of Biopredictive, the company marketing FibroTest-SteatoTest.

## Authors' contributions

TP conceived and wrote the manuscript. TP, VR, SN, DT, JM, MM, MM and JA were responsible for the patient drafting, and participated in the coordination of the study. FIB, AA and DM carried out biochemical analysis and drafted the paper. FC and DC were responsible of histological analysis. TP performed the statistical analysis. All authors read and approved the final manuscript.
